# New HDAC6-mediated deacetylation sites of tubulin in the mouse brain identified by quantitative mass spectrometry

**DOI:** 10.1038/srep16869

**Published:** 2015-11-19

**Authors:** Ningning Liu, Yun Xiong, Shanshan Li, Yiran Ren, Qianqian He, Siqi Gao, Jun Zhou, Wenqing Shui

**Affiliations:** 1 State Key Laboratory of Medicinal Chemical Biology, College of Life Sciences, Nankai University, Tianjin 300071, China; 2 Key Laboratory of Systems Microbial Biotechnology, Tianjin Institute of Industrial Biotechnology, Chinese Academy of Sciences, Tianjin 300308, China

## Abstract

The post-translational modifications (PTMs) occurring on microtubules have been implicated in the regulation of microtubule properties and functions. Acetylated K40 of α-tubulin, a hallmark of long-lived stable microtubules, is known to be negatively controlled by histone deacetylase 6 (HDAC6). However, the vital roles of HDAC6 in microtubule-related processes such as cell motility and cell division cannot be fully explained by the only known target site on tubulin. Here, we attempt to comprehensively map lysine acetylation sites on tubulin purified from mouse brain tissues. Furthermore, mass spectrometry-based quantitative comparison of acetylated peptides from wild-type *vs* HDAC6 knockout mice allowed us to identify six new deacetylation sites possibly mediated by HDAC6. Thus, adding new sites to the repertoire of HDAC6-mediated tubulin deacetylation events would further our understanding of the multi-faceted roles of HDAC6 in regulating microtubule stability and cellular functions.

Microtubules, a major component of the cytoskeleton, are known to carry a plethora of post-translational modifications (PTMs) which constitute the tubulin code^1^. Analogous to the “histone code” that has been proposed to coordinate chromatin function and gene activity^2^,^3^,^4^, the tubulin code may contribute to microtubule-based functions by modulating microtubule interactions with diverse effectors^5^,^6^,^7^. Distinct from most tubulin-specific PTMs occurring on the unstructured carboxy-terminal tails (CTTs)^8^, acetylation is present on multiple lysine residues throughout the polypeptide chain of tubulin and it is functionally related to the stability and various activities of microtubules^9^,^10^,^11^,^12^. A prominent example is acetylated K40 of α-tubulin which resides inside the microtubule lumen^13^,^14^,^15^ and serves as a hallmark of long-lived and stable microtubules^10^. However, tubulin acetylation has not been verified to directly promote stabilization of microtubules^9^,^16^,^17^,^18^. Previous studies also demonstrated that K40 acetylation fosters the ability of kinesin-1 binding to microtubules yet does not govern motility of the motor kinesin-1^11^,^12^,^19^,^20^. In addition, acetylation of K252 on β-tubulin is reported to modulate microtubule polymerization in the cell^21^. Tubulin acetylation has been implicated in numerous cellular activities, such as the ATPase activity of the Na^+^/K^+^ pump^22^, ER sliding^23^ and mitochondrial fission^24^. Moreover, this particular tubulin PTM is speculated to play a role in neurodegenerative diseases such as Huntington’s disease and Parkinson’s disease^12^,^25^,^26^.

Proteomic surveys and biochemical studies have reported an array of acetylated lysine residues on α-/β-tubulin in mouse and human cells (Fig. 1A,B). Although acetylation seems to be an abundant PTM of tubulin^10^,^27^, its functional roles and upstream regulatory enzymes are only starting to be unravelled. Candidate acetyltransferases for α-tubulin K40 include MEC17 (Caenorhabditis elegans protein mechanosensory abnormality 17)^28^,^29^, ARD1–NAT1(arrestdefective 1–aminoterminal, α-amino, acetyltransferase 1)^30^, ELP complex (elongator protein complex)^30^,^31^ and GCN5 (general control of amino acid synthesis 5)^32^. Genetic knockout of MEC17 in mice led to hyperacetyled tubulin and a grossly normal phenotype^33^,^34^. Acetylation of K252 on β-tubulin is carried out by the acetyltransferase SAN^21^. On the other hand, two widely studied deacetylases targeting K40 are the class II histone deacetylase HDAC6 (histone deacetylase 6)^35^,^36^,^37^, and the class III NAD-dependent histone deacetylase SIRT2 (sirtuin 2)^38^. HDAC6 overexpression resulted in significant deacetylation of microtubules, whereas inhibition of HDAC6 or SIRT2 increased microtubule acetylation levels and altered cell movement and survival^5^,^35^,^36^,^37^,^38^,^39^. However, for most other documented tubulin acetylation sites, their connections to annotated acetyltransferases or deacetylases remain unknown.

HDAC6 is an intensively studied deacetylase located mainly in the cytoplasm. It is regarded as a promising therapeutic target because of its implications in neurodegenerative disorders, immune activities and depressive behaviors^40^,^41^,^42^,^43^. Its notable substrates are α-tubulin^35^,^37^, Hsp90^44^,^45^, cortactin^46^, peroxiredoxins^47^. The growing list of new substrates identified for this enzyme^48^,^49^,^50^,^51^,^52^ would facilitate deciphering the precise roles of HDAC6 involved in various cellular processes such as cell motility^35^,^46^,^53^,^54^, cell survival^50^,^51^, redox homeostasis^47^ and stress response^55^. As to tubulin, the first identified substrate of HDAC6, it is possible that HDAC6-mediated deacetylation occurs not only on K40 of α-tubulin^56^, given that a number of lysine residues located on the microtubule wall might be easier for HDAC6 to access than K40 which resides in the microtubule lumen. In addition, HDAC6 might deacetylate β-tubulin given that β-tubulin is highly homologous to α-tubulin and known to undergo acetylation and interacts with HDAC6 both *in vitro* and *in vivo*^37^.

In the present study, we applied a quantitative mass spectrometry approach to identify putative HDAC6-mediated deacetylation sites on tubulin purified from mouse brain tissues^57^,^58^. Comparison of acetylation abundances on specific sites of tubulin between the wild-type and HDAC6 knockout mice revealed that tubulin acetylation regulated by HDAC6 is not restricted to K40 of α-tubulin. Our findings would help infer a comprehensive role of HDAC6 in mediating multiple microtubule-based processes.

## Results

### Mapping lysine acetylation sites in tubulin isolated from mouse brain tissues

**Figure 2:**
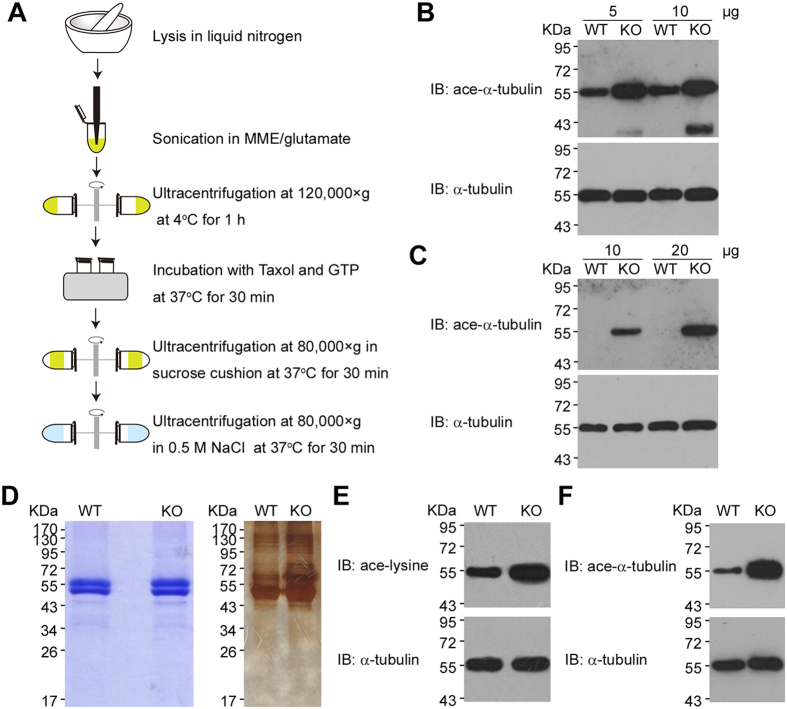
Isolation and characterization of mouse brain tubulin. (**A**) Schematic diagram of tubulin isolation from brain tissues in mice. It was drawn by author Ningning Liu. (**B**,**C**) Immunoblots of tubulin isolated from brain tissues (**B**) or heart tissues (**C**) in wild-type and HDAC6 knockout mice to analyze K40 acetylation in α-tubulin. (**D**) Coomassie blue staining and silver staining of tubulin isolated from brain tissues in wild-type and HDAC6 knockout mice. (**E**,**F**) Immunoblots of tubulin isolated from brain tissues to analyze pan-acetylation (**E**) and K40 acetylation in α-tubulin (**F**).

To identify all possible lysine acetylation sites in tubulin, we first isolated tubulin from the brain tissue in the wild-type (WT) and HDAC6 knockout (KO) mice using a modified protocol based on Taxol-induced microtubule polymerization and ultracentrifugation^59^ (Fig. 2A). The brain tissue was chosen here as the source for the purification of acetylated tubulin because tubulin is known to be highly expressed in this tissue^60^. In our experiment, more than 100 μg tubulin was obtained from 100 mg brain tissues in contrast to less than 40 μg tubulin from the same mass of cell extracts. In addition, immunoblots with an ace-K40-specific antibody showed strong signals in the brain tissue extracts from both WT and KO mice compared to the heart tissue extracts (Fig. 2B,C), suggesting a better chance for comprehensive acetylation site mapping of tubulin from the mouse brain.

Relatively high purity of isolated tubulin was shown by SDS-PAGE stained with Coomassie blue, though we noticed that other proteins probably interacting with tubulin were co-purified in low amount as indicated by more sensitive silver stain (Fig. 2D). It is noteworthy that two distinct protein bands with the molecular weight close to 55 KDa were present in the isolated tubulin. They turned out to be mainly α-tubulin (upper band) and β-tubulin (lower band) by mass spectrometry analysis of the in-gel protein digests. Immunoblots on isolated tubulin clearly verified the increase of both pan-acetylation and K40 acetylation levels in KO *vs* WT mouse tissue (Fig. 2E,F).

**Figure 3:**
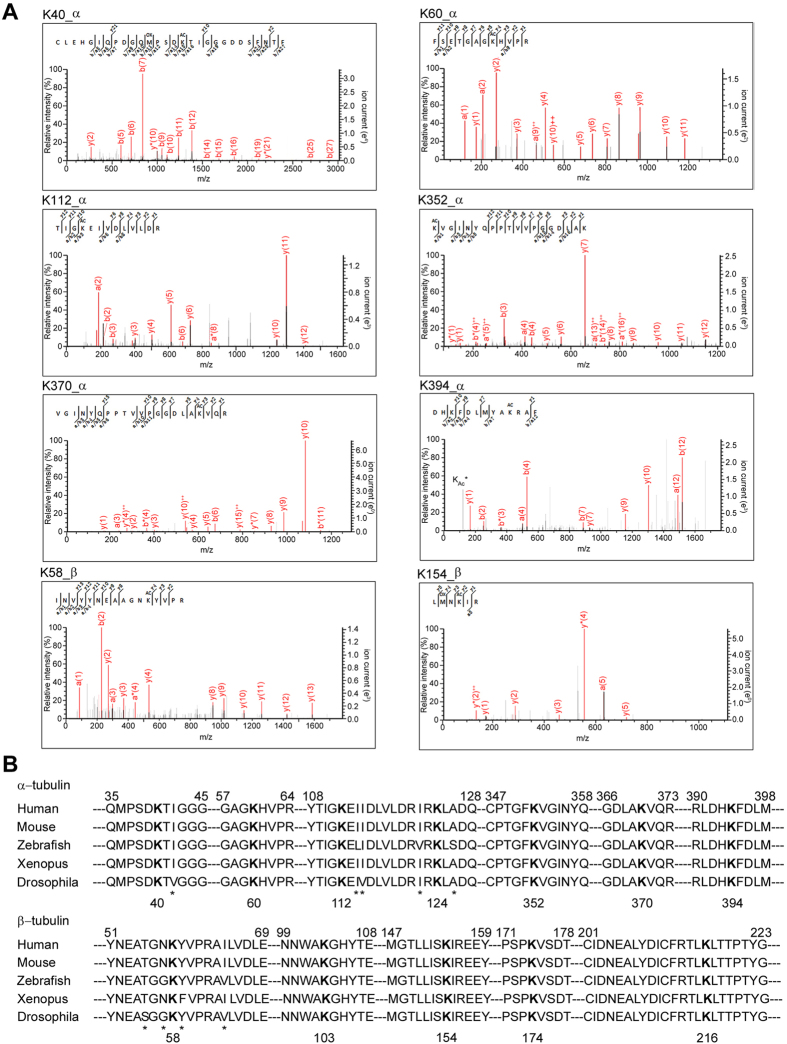
Tandem mass spectrometry spectra and sequence alignment of identified tubulin acetylation sites. (**A**) Tandem mass spectrometry spectra of selected α-tubulin or β-tubulin peptides with acetylated (ac) lysine residues. The acetyl lysine ammonium ion was marked by K_AC_^*^. (**B**) Alignment of α-tubulin or β-tubulin sequences of human and other organisms. Residues not conserved are marked with asterisks. Acetylated lysine residues identified in this study are in bold.

The two tubulin bands from either KO or WT mouse brain were separately digested and resulting peptide mixtures were analyzed by high-resolution mass spectrometry. Our analysis identified twelve lysine acetylation residues with stringent criteria, among them seven are new sites not documented for mouse tubulin in UniProt protein database. Representative MS/MS spectra for eight tubulin peptides acetylated on different sites including four new ones are shown in Fig. 3A, and the other four are listed in Supplementary Fig. S1. Notably, all of these acetylation residues are highly conserved across the α-tubulin and β-tubulin sequences in various organisms from drosophila to human (Fig. 3B), implying their possibly prominent roles retained in evolution.

### Putative tubulin deactylation sites mediated by HDAC6

**Figure 4:**
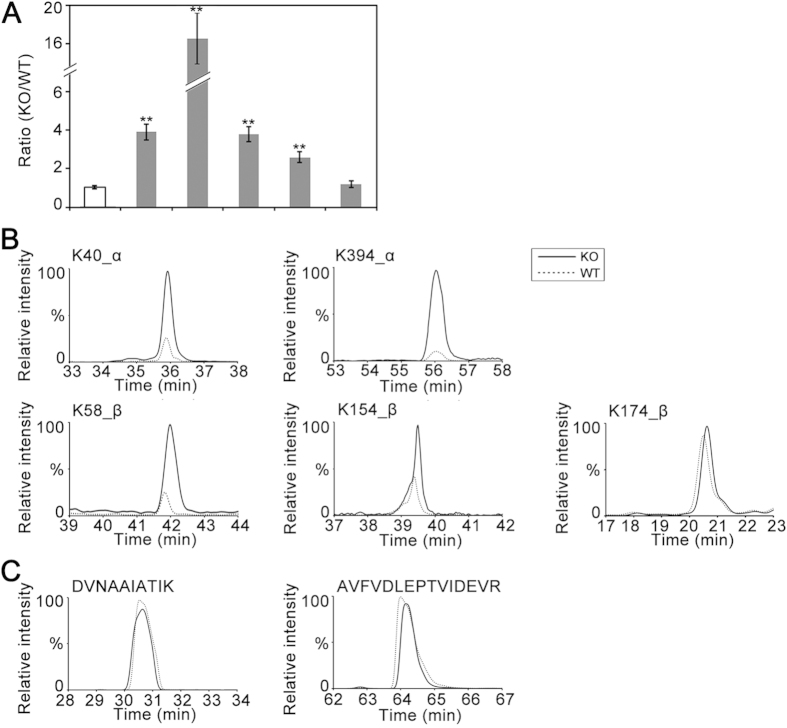
Quantitative comparison of acetylated tubulin peptides between HDAC6 KO and WT mice. (**A**) Summary of relative ratios of acetylated tubulin peptides detected from HDAC6 KO *vs* WT mice. The average ratio of unmodified tubulin peptides is the control. Significant changes of site-specific acetylation in HDAC6 KO *vs* WT mice are indicated by asterisks (*p*-value < 0.01). Each ratio is the mean value from experimental triplicates, and error bars indicate SD. (**B**) The extracted ion chromatograms of specific acetylated peptides from HDAC6 KO and WT mice. (**C**) The extracted ion chromatograms of two representative unmodified peptides from HDAC6 KO and WT mice. The peptide sequences are shown above the graph.

In addition to mapping acetylation sites in isolated tubulin, we employed a quantitative MS approach to search for lysine residues in tubulin specifically mediated by HDAC6. To this end, individual acetylated tubulin peptides and unmodified tubulin peptides were quantified by their MS responses. As the tubulin expression measured by unmodified peptide responses was unchanged in KO *vs* WT samples, relative variation of modified peptide responses would reflect regulation of site-specific acetylation as a result of HDAC6 deficiency. The relative ratios of five acetylated peptides as well as the average ratios of several unmodified peptides from α-/β-tubulin in KO *vs* WT mice are summarized in Fig. 4A, and their MS response curves are shown in Fig. 4B,C. The acetylation level of a specific lysine is considered significantly changed between KO and WT samples if its corresponding peptide ratio is statistically varied from the average unmodified peptide ratio (Fig. 4A). As expected, the peptide containing acetylated K40 known to be targeted by HDAC6 was up-regulated by almost 4-fold in KO mice (Fig. 4B). By contrast, for K174 of β-tubulin, its acetylation level was not perturbed under HDAC6 depletion. The rest of three peptides all increased the acetylation abundance in KO vs WT samples, thus suggesting that their lysine residues (K394 of α-tubulin and K58, K154 of β-tubulin) are putative sites mediated by HDAC6. Moreover, the exclusive detection of acetylated K60 and K370 in α-tubulin peptides and acetylated K103 in β-tubulin peptides indicated deacetylation of the three lysine residues are dominantly controlled by HDAC6. Constant tubulin expression was demonstrated by equivalent MS responses of two unmodified tubulin peptides (Fig. 4C).

### Distribution of identified lysine acetylation sites in the tubulin structure

**Figure 5:**
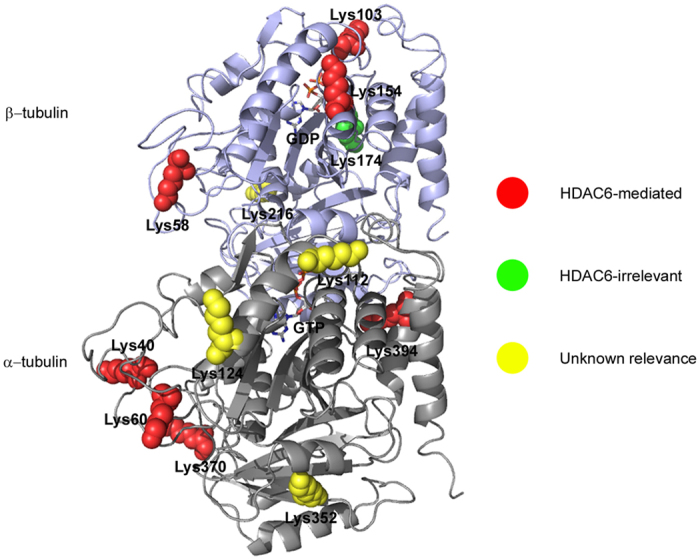
Distribution of identified lysine acetylation sites in the mouse tubulin structure. Red and green spheres mark sites that are mediated by HDAC6 and unrelated to HDAC6 activity, respectively. Yellow spheres are sites with unknown relevance to HDAC6 because the related peptides cannot be quantified.

We then marked all lysine acetylation sites identified in this study in the 3D structure of α-/β-tubulin dimer (accession number PDB#1TUB)^61^ (Fig. 5). They are classified into HDAC6-mediated, HDAC6-unrelated or sites with unknown relevance to HDAC6. Interestingly, our study reported three acetylation sites (K60, K370 of α-tubulin, K58 of β-tubulin) presumably located in the lumen of microtubules in addition to the widely-recognized luminal modification site K40^13^,^14^,^15^,^62^,^63^, and all of them are possibly mediated by HDAC6. HDAC6 seems to be also capable of regulating the acetylation of K394 on α-tubulin exposed to the surface of the microtubule. Among the five sites located at the interface of α- and β-tubulin subunits, K103 and K154 of β-tubulin are possibly targeted by HDAC6. Therefore, HDAC6 may implement a complex mechanism to access multiple lysine residues at different locations of the microtubule for deacetylation.

## Discussion

Our study mapped twelve acetylation sites of α-/β- tubulin isolated from the mouse brain including seven new sites not documented in UniProt protein database. Furthermore, six lysine residues were revealed to be putative targets of HDAC6, in addition to K40 which is so far the only known deacetylation site of HDAC6. Thus, adding new sites to the repertoire of HDAC6-mediated deacetylation events on tubulin would further our understanding of the multi-faceted roles of HDAC6 in regulating microtubule stability and cellular functions.

Our established experimental method combining tubulin isolation and quantitative mass spectrometry analysis can be extended to characterization of HDAC6-mediated tubulin deacetylation in tissues other than the brain. Earlier studies reported high expression of HDAC6 transcripts in heart, liver, kidney and pancreas^64^. Therefore, it is worthy of future efforts to profile tubulin deacetylation sites in different tissues targeted by HDAC6, which would shed lights on tissue-specific functions of HDAC6.

It is of our notion that the methodology adopted in this study is unable to pinpoint lysine residues in tubulin that are directly targeted by HDAC6. Chances are that acetylation of these residues could be controlled by other deacetylases or acetyltrasferases for which the activities were perturbed as a consequence of HDAC6 knockout. To distinguish the direct and indirect effect of HDAC6 on site-specific deacetylation of tubulin, it is necessary to perform an *in vitro* deacetylation experiment using purified HDAC6 and acetylated tubulin peptides.

Interestingly, in our mass spectrometry quantification of acetylation levels of different tubulin peptides, we examined the abundances of a few unmodified peptides from tubulin in the wild-type and HDAC6 KO mice. They all showed equivalent MS responses, suggesting that the tubulin protein levels were not significantly perturbed due to HDAC6 depletion (Fig. 4C). We also assessed tubulin expression in the mutants of K394 (α-tubulin) and K58 (β-tubulin) by immunoblotting. For K394, the expression of its acetyl-deficient and acetyl -mimic mutants was largely reduced compared to the wild-type; for K58, the tubulin expression was constant across mutants and the wild-type (Figure S2).

Three new lysine residues (K60, K370 of α-tubulin, K58 of β-tubulin) in the lumen of microtubules are found to be possible targets of HDAC6, apart from the widely studied K40. It still remains elusive how the acetylation-mediated enzymes get access to the luminal residues. Some hypotheses have been proposed that HDAC6 may exploit a mechanism similar to MEC17 (α-tubulin acetyltransferase) which approaches K40 either by diffusing along the microtubule lumen or entering through holes that exist in the microtubule lattice^28^,^29^. Alternatively, HDAC6 might be recruited to α-tubulin subunits in the microtubule by first interacting with β-tubulin in a manner resembling the binding of certain drugs to microtubules^8^,^15^,^35^. Our finding of multiple residues as the putative targets of HDAC6 may indicate a complex and unique mechanism of HDAC6-mediated deacetylation.

## Materials and Methods

### Mice

HDAC6 knockout (KO) mice were in 129/C57BL6 mixed genetic background, generated and genotyped according to the previously described^65^. HDAC6 heterozygous mice were obtained from Tso-Pang Yao (Duke University, School of Medicine) and intercrossed to generate HDAC6 KO and WT littermates. Adult female mice (2 months old) were used for tubulin isolation. All mouse experiments are carried out in accordance with the relevant institutional and national guidelines and regulations, approved by the Animal Care and Use Committee of Nankai University, and conform to the relevant regulatory standards.

### Antibodies

For immunoblotting, antibodies against α-tubulin, acetylated α-tubulin (6-11B-1) and acetylated lysine were from Abcam, Sigma-Aldrich and Cell Signaling Technology, respectively. The peroxidase-conjugated secondary antibodies (Amersham Biosciences) were used.

### Immunoblotting

Proteins were resolved by SDS-PAGE and transferred onto polyvinylidenedifluoride membranes (Millipore). The membranes were blocked and incubated with primary antibodies and then horseradish peroxidase-conjugated secondary antibodies. Specific proteins were visualized with enhanced chemiluminescence detection reagent (Pierce Biotechnology).

### Taxol-based isolation of tubulin from mouse brains

A published protocol for tubulin purification^59^ was adopted with minor modifications. Brain tissues were reduced to a powder in a mortar in the presence of liquid nitrogen. The suspension of brain tissues in MME/glutamate buffer MME buffer [0.1 M 2-(N-morpholino) ethanesulfonic acid (pH 6.9), 1 mM MgCl_2_, 1 mM EGTA, and 1 M glutamate ] contained protease inhibitors and DTT were sonicated with a microtip probe (Branson, Danbury, CT) for 2 min on ice with 1 s on and 2 s off. Then samples were spun at 30,000 × g (Beckman TL100 centrifuge) for 15 min at 4 °C to discard cell debris, followed by centrifugation at 120,000 g at 4 °C for 1 h. The supernatants were transferred to 1.5-ml tubes and incubated for 30 min at 37 °C in the presence of 20 μM Taxol and 1 mM GTP, then centrifuged at 80,000 × g (Beckman TL100 centrifuge) for 30 min at 37 °C on 300 μl cushion containing 20% sucrose, 20 μM Taxol, and 1 mM GTP. Supernatants were resuspended and incubated for 20 min at 37 °C in 1 ml of MME/glutamate buffer containing 0.5 M NaCl and 20 μM Taxol. Microtubules were pelleted by centrifugation at 80,000 × g for 30 min at 37 °C, snap frozen and stored at −80 °C.

### Tubulin digestion

The purified proteins were loaded onto SDS-PAGE. The two bands around 55 KDa were excised from the gel and subjected to in-gel tryptic digestion according to the procedure previously described^66^. The digested peptides were extracted and then evaporated in a speed vacuum apparatus.

### NanoLC-MS/MS analysis

Peptide samples were analyzed by reverse-phase liquid chromatography-electrospray ionization-MS/MS using an Eksigent Ultra Plus nano-LC 2D HPLC system connected to a quadrupole time of flight TripleTOF 5600 mass spectrometer (AB SCIEX). The vacuum dried peptides were redissolved in 0.1% formic acid solution and then loaded to the trapping column with a flow rate of 5 μl/min for 20 min. Thereafter, peptides mixtures were loaded onto the trapping column and then separated in the analytical C18-nano-cappillary LC column (100 μm ID × 10 cm, 3 μm particle size; New Objective). A 70-min gradient from 2 to 35% B (mobile phase A = 2% acetonitrile/98% of 0.1 formic acid in H_2_O; mobile phase B = 98% acetonitrile/2% of 0.1% formic acid in H_2_O) at a flow rate of 300 nL/min was adopted for peptide separation. MS analysis was performed in positive ion mode and high-sensitivity mode. The system was tuned for a minimum resolution of ~35,000 full-width half-maximum in MS1 and 15,000 in MS2. Data acquisition was performed using Analyst 1.5.1 (AB SCIEX) with an ion spray voltage of 2.4 kV, curtain gas of 30 psi, ion source gas of 4 psi, and interface heater temperature of 150 °C. The m/z range for MS and MS/MS scans was set from 350 to 1500 and from 100 to 1500, respectively. After acquisition of 4 samples, TOF MS spectra and TOF MS/MS spectra were automatically calibrated during dynamic LC-MS and MS/MS autocalibration acquisitions by injecting β-galactosidase. The mass window for precursor ion selection of the quadrupole mass analyzer was set to ±1 *m/z*. For MS/MS collection, both IDA and targeted precursor strategies were adopted. In the IDA mode, the MS/MS spectra of the 20 most abundant parent ions were obtained following each survey MS1 scan (allowing typically for 50 ms acquisition time per each MS/MS). Dynamic exclusion mass width was set to be 50 mDa and exclusion duration to be 18 s. For the target precursor strategy, only the MS/MS spectra of selected acetylated peptide ions with defined m/z values were obtained and the acquisition time per targeted MS/MS was 150 ms. For quantitative comparison, tubulin digestion and mass spectrometry analysis (IDA mode) of each sample was performed in triplicate.

### Data processing for identification and quantification of peptide acetylation

The raw MS data was processed using Mascot server version 2.3.02 (Matrix Sciences). For protein identification, a Uniprot database consisting of all human protein sequences was employed. The parameters were set as follows: peptide mass tolerance, 15 ppm; missed cleavages, 4; fixed modification, carbamidomethylation of cysteine; variable modification, oxidation of methionine and acetylation of lysine/arginine; threshold of peptide false discovery rate (FDR) by reverse database search, 1.0%. Identification of acetylated peptides had to meet the following stringent criteria: (1) acetylated peptides are identified with an expectation value <1% FDR; (2) the actual mass error of the precursor ion is below 10 ppm; (3) peptides with assigned acetylation sites on the C-terminal are removed; (4) the same acetylated peptides are identified in three biological replicates; (5) the unmodified peptide counterparts are also identified. A list of acetylated peptide sequences and mass spectrometry data is provided in SI Table S1.

For the confidently identified acetylated peptides, their MS responses were measured by the peak area of the corresponding extracted ion chromatograms based on the peptide precursor accurate mass with a tolerance of 5 mDa. The Peakview software (AB SCIEX) was used for the peptide ion extraction and peak area calculation. The MS responses of acetylated peptides from the HDAC6 knockout mice were then compared to those from the wild type mice to derive the relative ratios. Then the ratios of acetylated peptides were statistically compared with the average ratios of unmodified peptides to determine whether acetylation levels of specific sites are significantly changed in HDAC6 knockout mice *vs* the wild type.

## Additional Information

**How to cite this article**: Liu, N. *et al.* New HDAC6-mediated deacetylation sites of tubulin in the mouse brain identified by quantitative mass spectrometry. *Sci. Rep.* **5**, 16869; doi: 10.1038/srep16869 (2015).

## Supplementary Material

Supplementary Information

## Figures and Tables

**Figure 1:**
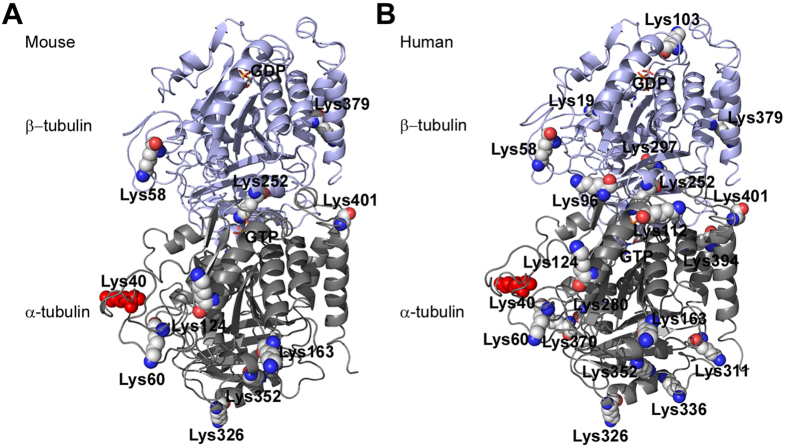
Reported acetylation sites in the 3D structure of mouse tubulin (A) and human tubulin (B). K40 of α-tubulin is shown with the red sphere. GTP bound to α-tubulin and GDP bound to β-tubulin are shown as colored sticks.
