# Supplementary material for: New HDAC6-mediated deacetylation sites of tubulin in the mouse brain identified by quantitative mass spectrometry
Source: Sci Rep. 2015 Nov 19;5:16869. doi: 10.1038/srep16869 (PMC4652237; doi:10.1038/srep16869)
Supplement: Supplementary Information [file srep16869-s1.doc]

**Supporting Information for Manuscript**

New HDAC6-mediated deacetylation sites of tubulin in the mouse brain identified by quantitative mass spectrometry

Ningning Liu^1#^, Yun Xiong^2#^, Shanshan Li^2^, Yiran Ren^1^, Qianqian He^1^, Siqi Gao^1^, Jun Zhou^1^*, Wenqing Shui^2*^


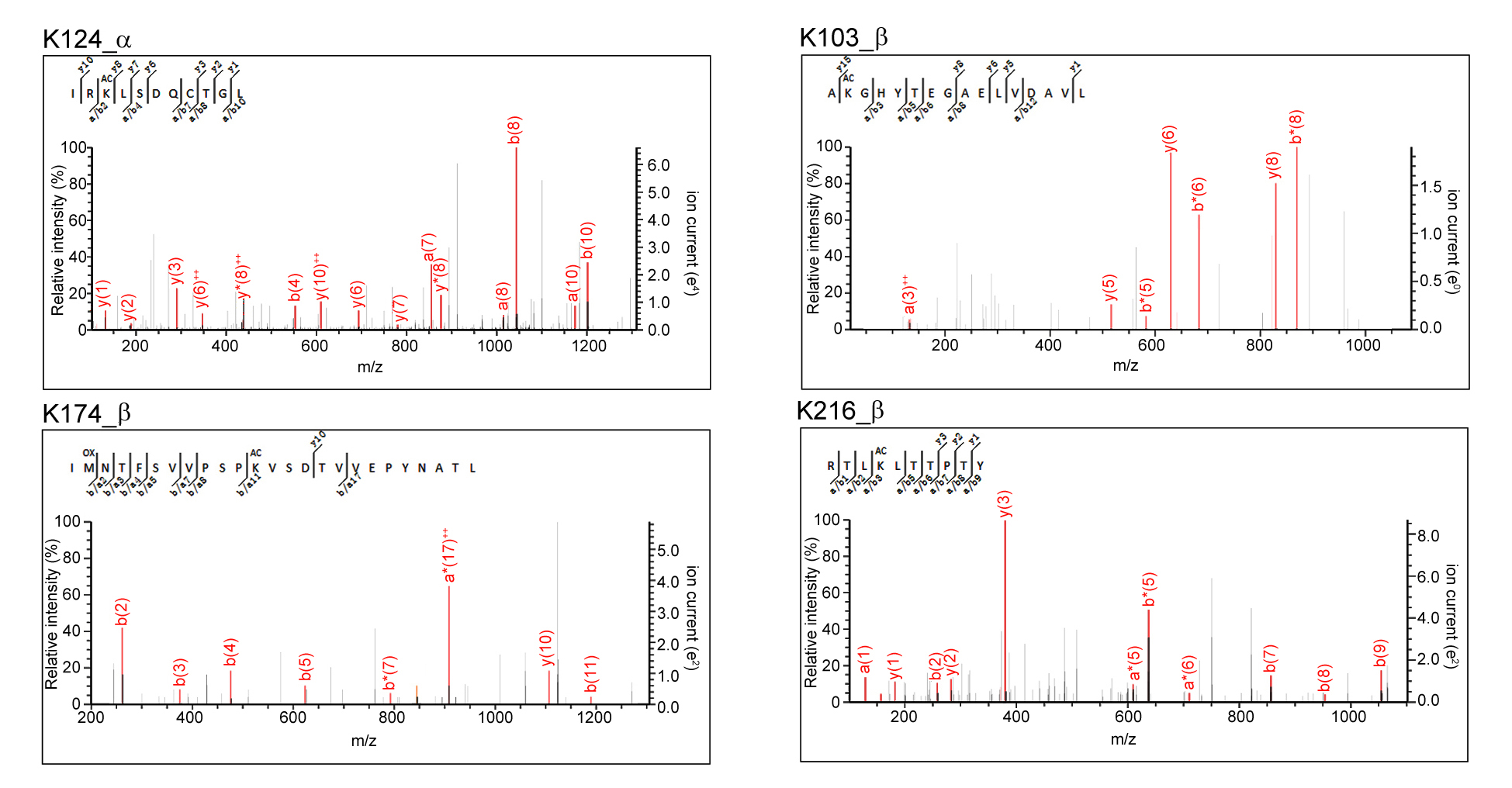


Supplementary Figure S1. Tandem mass spectrometry spectra of the rest of acetylated peptides from α-tubulin (A-B) or β-tubulin (C-E) identified in this study. "The rest" refers to tubulin acetylation sites identified in our study other than those shown in Figure 3A.


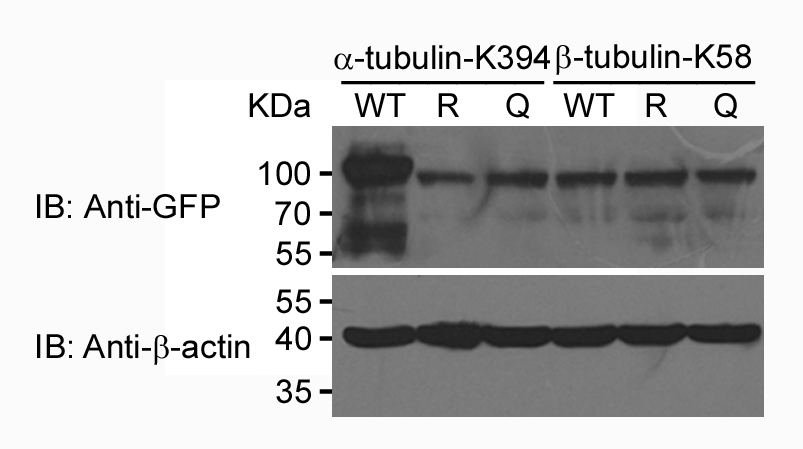


Supplementary Figure S2. Immunoblot analysis of GFP-α-tubulin wild-type and mutants (K394R and K394Q), GFP-β-tubulin wild-type and mutants (K58R and K58Q) in 293T cells.

Table S1. Summary of acetylated peptides of tubulin from mouse brain identified in this study

| **Protein name** | **m/z obs** | **Mr obs** | **Charge** | **Mr Calc.** | **Delta mass** | **Error (ppm)** | **Stard** | **End** | **Expect value** | **Peptide sequence (acetyl-K in red)** | **Mod site** |
| --- | --- | --- | --- | --- | --- | --- | --- | --- | --- | --- | --- |
| Tubulin alpha-1A | 1027.781 | 3080.3205 | 3 | 3080.3128 | 0.0077 | 2.5 | 25 | 52 | 4.90E-10 | CLEHGIQPDGQMPSDKTIGGGDDSFNTF | K40 |
| Tubulin alpha 1C | 664.3414 | 1326.6683 | 2 | 1326.668 | 0.0003 | 0.2 | 53 | 64 | 1.30E-10 | FSETGAGKHVPR | K60 |
| Tubulin alpha-3 | 756.9287 | 1511.8429 | 2 | 1511.8559 | -0.013 | -8.6 | 109 | 121 | 2.60E-04 | TIGKEIVDLVLDR | K112 |
| Tubulin alpha-4A | 666.8537 | 1331.6929 | 2 | 1331.6867 | 0.0062 | 4.7 | 151 | 161 | 5.00E-03 | IRKLSDQCTGL | K124 |
| Tubulin alpha-1A | 665.7034 | 1994.0883 | 3 | 1994.0837 | 0.0046 | 2.3 | 352 | 370 | 1.20E-03 | KVGINYQPPTVVPGGDLAK | K352 |
| Tubulin alpha 1C | 750.7389 | 2249.195 | 3 | 2249.2168 | -0.0218 | -9.7 | 353 | 373 | 2.40E-04 | VGINYQPPTVVPGGDLAKVQR | K370 |
| Tubulin alpha-1B | 561.9519 | 1682.8329 | 3 | 1682.8239 | 0.009 | 5.3 | 392 | 404 | 8.40E-03 | DHKFDLMYAKRAF | K394 |
| Tubulin beta-2A | 638.3187 | 1911.9342 | 3 | 1911.9479 | -0.0137 | -7.2 | 47 | 62 | 3.10E-05 | INVYYNEAAGNKYVPR | K58 |
| Tubulin beta-4A | 572.2883 | 1713.8425 | 3 | 1713.8573 | -0.0148 | -8.6 | 102 | 117 | 9.20E-03 | AKGHYTEGAELVDAVL | K103 |
| Tubulin beta-1B | 416.7368 | 831.459 | 2 | 831.4636 | -0.0046 | -5.5 | 151 | 156 | 4.50E-03 | LMNKIR | K154 |
| Tubulin beta-4B | 692.3552 | 2765.3919 | 4 | 2765.3834 | 0.0085 | 3.1 | 163 | 187 | 3.80E-03 | IMNTFSVVPSPKVSDTVVEPYNATL | K174 |
| Tubulin beta-4B | 618.3575 | 1234.7006 | 2 | 1234.6921 | 0.0085 | 6.9 | 213 | 222 | 7.00E-03 | RTLKLTTPTY | K216 |
